# Malignant Mesothelioma Mortality — United States, 1999–2015

**DOI:** 10.15585/mmwr.mm6608a3

**Published:** 2017-03-03

**Authors:** Jacek M. Mazurek, Girija Syamlal, John M. Wood, Scott A. Hendricks, Ainsley Weston

**Affiliations:** ^1^Respiratory Health Division, National Institute for Occupational Safety and Health, CDC; ^2^Division of Safety Research, National Institute for Occupational Safety and Health, CDC.

Malignant mesothelioma is a neoplasm associated with occupational and environmental inhalation exposure to asbestos* fibers and other elongate mineral particles (EMPs) (*1*–*3*). Patients have a median survival of approximately 1 year from the time of diagnosis (*1*). The latency period from first causative exposure to malignant mesothelioma development typically ranges from 20 to 40 years but can be as long as 71 years (*2*,*3*). Hazardous occupational exposures to asbestos fibers and other EMPs have occurred in a variety of industrial operations, including mining and milling, manufacturing, shipbuilding and repair, and construction (*3*). Current exposures to commercial asbestos in the United States occur predominantly during maintenance operations and remediation of older buildings containing asbestos (*3*,*4*). To update information on malignant mesothelioma mortality (*5*), CDC analyzed annual multiple cause-of-death records^†^ for 1999–2015, the most recent years for which complete data are available. During 1999–2015, a total of 45,221 deaths with malignant mesothelioma mentioned on the death certificate as the underlying or contributing cause of death were reported in the United States, increasing from 2,479 deaths in 1999 to 2,597 in 2015 (in the same time period the age-adjusted death rates^§^ decreased from 13.96 per million in 1999 to 10.93 in 2015). Malignant mesothelioma deaths increased for persons aged ≥85 years, both sexes, persons of white, black, and Asian or Pacific Islander race, and all ethnic groups. Despite regulatory actions and the decline in use of asbestos the annual number of malignant mesothelioma deaths remains substantial. The continuing occurrence of malignant mesothelioma deaths underscores the need for maintaining measures to prevent exposure to asbestos fibers and other causative EMPs and for ongoing surveillance to monitor temporal trends.

For this report, malignant mesothelioma deaths during 1999–2015 were identified from death certificates and included deaths for which *International Classification of Diseases (ICD)*, *10th Revision* codes for malignant mesothelioma^¶^ were listed as either the underlying or contributing cause of death in the multiple cause-of-death mortality data. The analysis was restricted to deaths of persons aged ≥25 years, as they were more likely to have been occupationally exposed than were younger decedents. Age-adjusted death rates per 1 million persons aged ≥25 years by demographics, neoplasm anatomical site, and year were calculated using the 2000 U.S. Census standard population estimate. Industry and occupation information was available from death certificates for decedents reported from 23 states for 1999, 2003, 2004, and 2007, and was coded** using the U.S. Census 2000 Industry and Occupation Classification System. Proportionate mortality ratios (PMRs)^††^ for malignant mesothelioma by industry and occupation were calculated. Confidence intervals (CIs) were calculated assuming Poisson distribution of the data.

During 1999–2015, a total of 45,221 deaths with malignant mesothelioma mentioned on the death certificate as the underlying or contributing cause of death among persons aged ≥25 years were reported in the United States; 16,914 (37.4%) occurred among persons aged 75–84 years, 36,093 (79.8%) occurred among males, 42,778 (94.6%) among whites, and 43,316 (95.8%) among non-Hispanics (Table 1). Malignant mesothelioma was classified as mesothelioma of pleura (3,351; 7.4%), peritoneum (1,854; 4.1%), pericardium (74; 0.2%), other anatomic site (5,280; 11.7%), and unspecified anatomic site (35,068; 77.5%). Among 42,470 (93.9%) decedents, malignant mesothelioma was coded as the underlying^§§^ cause of death.

**TABLE 1:** Malignant mesothelioma deaths and age-adjusted rates* among decedents aged ≥25 years, by selected characteristics — United States, 1999–2015

Characteristics	No. of deaths	Death rate
**Total**	**45,221**	**13.10**
Underlying^†^ cause	42,470	12.30
Age group (yrs)^§^		
25–34	138	0.20
35–44	544	0.75
45–54	1,936	2.69
55–64	6,237	11.22
65–74	12,985	36.31
75–84	16,914	76.28
≥85	6,467	74.46
Sex		
Male	36,093	24.94
Female	9,128	4.65
Race		
White	42,778	14.25
Black or African American	1,870	5.84
Asian or Pacific Islander	440	3.52
American Indian or Alaska Native	133	5.96
Ethnicity		
Hispanic	1,815	7.38
Non-Hispanic	43,316	13.46
Unknown	90	—
Anatomic site^¶^		
Pleura	3,351	0.98
Peritoneum	1,854	0.51
Pericardium	74	0.01
Other	5,280	1.52
Unspecified	35,068	10.14
Year		
1999	2,479	13.96
2000	2,529	14.16
2001	2,504	13.77
2002	2,570	13.92
2003	2,621	13.95
2004	2,656	13.94
2005	2,701	13.93
2006	2,586	13.19
2007	2,603	12.98
2008	2,706	13.26
2009	2,752	13.20
2010	2,744	13.10
2011	2,829	13.16
2012	2,873	12.97
2013	2,686	11.80
2014	2,785	11.98
2015	2,597	10.93
P-value**	0.001	<0.001

* Age-adjusted death rates per 1 million persons calculated using the 2000 Standard population.

^†^ Underlying cause of death is defined as “the disease or injury which initiated the chain of morbid events leading directly to death, or the circumstances of the accident or violence which produced the fatal injury.”

^§^ Age-specific death rates per 1 million persons.

^¶^ The sum of anatomic site totals (45,627) is greater than the total number of deaths (45,221) because some decedents have more than one site listed on their death certificate.

** For 1999–2015, linear time trend was examined using a first-order autoregressive linear regression model to account for the serial correlation.

During 1999–2015, the annual number of malignant mesothelioma deaths increased 4.8% overall, from 2,479 in 1999 to 2,579 in 2015 (p-value for linear time trend <0.001). The number of malignant mesothelioma deaths increased among persons aged ≥85 years, both sexes, white, black, and Asian or Pacific Islander race, and all ethnic groups; and patients with mesothelioma of the peritoneum and unspecified anatomic site. Malignant mesothelioma deaths decreased among persons aged 35–44, 45–54, and 55–64 years, and among persons with mesothelioma of the pleura and other anatomic sites.

During 1999–2015, the mesothelioma age-adjusted death rate decreased 21.7% from 13.96 per million population (1999) to 10.93 (2015) (p-value for time trend <0.001). This trend in the standardized rate is a weighted average of the trends in the age-specific rates and masks the differences in individual age groups. The age-specific death rate decreased significantly among persons 45–54 (p<0.001), 55–64 (p<0.001), and 65–74 (p<0.001) years and increased significantly among persons aged ≥85 years (p<0.001). During 1999–2015, the annualized state mesothelioma age-adjusted death rate exceeded 20 per million per year in two states: Maine (22.06) and Washington (20.10) (Figure).

**FIGURE:**
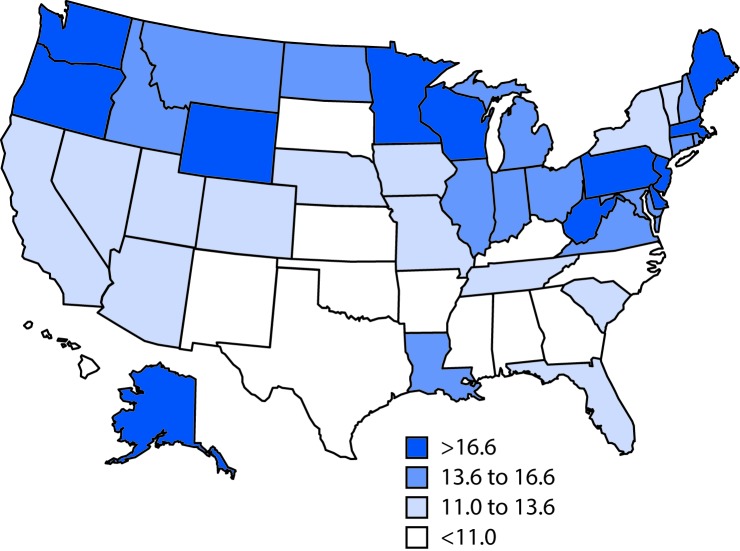
Malignant mesothelioma annualized age-adjusted death rate* per 1 million population,^†^ by state — United States, 1999–2015 * Age-adjusted death rates were calculated by applying age-specific death rates to the 2000 U.S standard population age distribution (https://wonder.cdc.gov/wonder/help/mcd.html#Age-Adjusted Rates). In two states (Maine and Washington), the age-adjusted death rate exceeded 20 per million per year. ^†^ Decedents aged ≥25 years for whom the *International Classification of Diseases, 10th Revision* codes C45.0 (mesothelioma of pleura), C45.1 (mesothelioma of peritoneum), C45.2 (mesothelioma of pericardium), C45.7 (mesothelioma of other sites), or C45.9 (mesothelioma, unspecified) were listed on death certificates were identified using CDC multiple cause-of-death data for 1999–2015.

Industry and occupation data were available for 1,830 (96.3%) of 1,900 malignant mesothelioma deaths that occurred in residents of 23 states during 1999, 2003, 2004, and 2007 (Table 2).^¶¶^ Among 207 industries and 274 occupations, significantly elevated PMRs for malignant mesothelioma were found for 11 industries and 17 occupations. By industry, the highest PMRs were for ship and boat building and repairing (6.7; 95% CI = 4.3–9.9); petroleum refining (4.1; CI = 2.6–6.0); and industrial and miscellaneous chemicals (3.8; CI = 2.9–5.0). By occupation, the highest PMRs were for insulation workers (26.9; CI = 16.2–42.0); chemical technicians (4.9; CI = 2.1–9.6); and pipelayers, plumbers, pipefitters, and steamfitters (4.8; CI = 3.7–6.1).

**TABLE 2:** Industries and occupations with significantly elevated proportionate mortality ratios, 1,830 malignant mesothelioma decedents aged ≥25 years — 23 states,* 1999, 2003, 2004, and 2007

Characteristic	No. of deaths	PMR^†^ (95% CI)
Industry		
Ship and boat building	24	6.7 (4.3–9.9)
Petroleum refining	25	4.1 (2.6–6.0)
Industrial and miscellaneous chemicals	58	3.8 (2.9–5.0)
Labor unions	7	3.7 (1.5–7.6)
Miscellaneous nonmetallic mineral product manufacturing	5	3.6 (1.2–8.4)
Electric and gas and other combinations	7	3.1 (1.3–6.5)
Water transportation	12	2.3 (1.2–3.9)
Electric power generation transmission and distribution	24	2.2 (1.4–3.3)
U.S. Navy	11	2.0 (1.0–3.6)
Architectural, engineering, and related services	23	1.9 (1.2–2.8)
Construction	280	1.6 (1.4–1.8)
Unknown	42	—
All other industries	1,312	—
Occupation		
Insulation workers	19	26.9 (16.2–42.0)
Chemical technicians	8	4.9 (2.1–9.6)
Pipelayers, plumbers, pipefitters, and steamfitters	67	4.8 (3.7–6.1)
Chemical engineers	12	4.0 (2.1–7.1)
Sheet metal workers	17	3.5 (2.0–5.5)
Sailors and marine oilers	5	3.4 (1.1–8.0)
Structural iron and steel workers	10	3.3 (1.6–6.0)
Millwrights	14	3.1 (1.7–5.2)
Stationary engineers and boiler operators	15	2.9 (1.6–4.8)
Electricians	53	2.8 (2.1–3.7)
Welding, soldering, and brazing workers	30	2.1 (1.4–3.0)
Construction managers	37	2.0 (1.4–2.8)
Engineers, all other	12	2.0 (1.0–3.5)
Mechanical engineers	14	1.9 (1.0–3.2)
First-line supervisors or managers of mechanics, installers, and repairers	27	1.8 (1.2–2.6)
Machinists	39	1.6 (1.1–2.1)
First-line supervisors or managers of production and operating workers	40	1.4 (1.0–2.0)
Unknown	49	—
All other occupations	1,362	—

**Abbreviations:** CI = confidence interval; PMR = proportionate mortality ratio.

* Multiple cause-of-death mortality files. https://webappa.cdc.gov/ords/norms-io14.html.

^†^ PMR is defined as the observed number of deaths with malignant mesothelioma in a specified industry/occupation, divided by the expected number of deaths with malignant mesothelioma. The expected number of deaths is the total number of deaths in industry or occupation of interest multiplied by a proportion defined as the number of malignant mesothelioma deaths in all industries and/or occupations, divided by the total number of deaths in all industries/occupations. The malignant mesothelioma PMRs were internally adjusted by five-year age groups, gender, and race. CIs were calculated assuming Poisson distribution of the data.

## Discussion

The annual number of malignant mesothelioma deaths is increasing, particularly among persons aged ≥85 years, most likely representing exposure many years ago. However, although malignant mesothelioma deaths decreased in persons aged 35–64 years, the continuing occurrence of mesothelioma deaths among persons aged <55 years suggests ongoing occupational and environmental exposures to asbestos fibers and other causative EMPs, despite regulatory actions by the Occupational Safety and Health Administration (OSHA)*** and the Environmental Protection Agency^†††^ aimed at limiting asbestos exposure. OSHA established a permissible exposure limit for asbestos of 12 fibers per cubic centimeter (f/cc) of air as an 8-hour time-weighted average in 1971. This initial permissible exposure limit was reduced to 5 f/cc in 1972, 2 f/cc in 1976, 0.2 f/cc in 1986, and 0.1 f/cc in 1994 (*6*). Although inspection data during 1979–2003 indicated a general decline in the proportion of samples exceeding designated occupational exposure limits, 20% of air samples collected in the construction industry in 2003 for compliance purposes exceeded the OSHA permissible exposure limit. Moreover, asbestos products remain in use, and new asbestos-containing products continue to be manufactured in or imported^§§§^ into the United States. Although most deaths from malignant mesothelioma in the United States are the result of exposures to asbestos 20–40 years prior, new cases might result from occupational exposure to asbestos fibers during maintenance activities, demolition and remediation of existing asbestos in structures, installations, and buildings if controls are insufficient to protect workers. The OSHA asbestos standard describes engineering and work practice controls (e.g., use of wet methods, local exhaust ventilation, and vacuum cleaners equipped with high-efficiency particulate air [HEPA] filters) during asbestos handling, mixing, removal, cutting, application, and cleanup and requires the use of respiratory protection if these controls are not sufficient to reduce employee exposure to levels at or below the permissible limit. Moreover, family members of workers engaged in activities placing them at risk for asbestos exposures also have the potential for exposure to asbestos (*3*). In addition, ongoing research is focusing on the potential nonoccupational and environmental exposures to asbestos fibers and other EMPs (e.g., erionite, a naturally occurring fibrous mineral that belongs to a group of minerals called zeolites), and nonmineral elongate particles (e.g., carbon nanotubes) to assess exposures and potential health risks (*7*,*8*).

Among the 96.3% of deaths in 23 states for which industry and occupation were known, shipbuilding and construction industries were major contributors to malignant mesothelioma mortality (*4*). The large number of deaths among construction workers is consistent with large number of construction workers with prior direct and indirect exposure to asbestos fibers through most of the 20th century (the construction industry accounted for 70%–80% of asbestos consumption) (*4*). For example, direct exposure to asbestos has occurred during installation of asbestos-cement pipes, asbestos-cement sheets, architectural panels, built-up roofing, and removal of roofing felts or asbestos insulation. Workers also might have been exposed to asbestos during spraying of asbestos insulation in multistoried structures during 1958–1972 (asbestos-containing materials were banned for fireproofing/insulating in 1973) (*4*). In addition, workers in other occupations (e.g., carpenters, electricians, pipefitters, plumbers, welders) might also have been exposed if they were present on-site during spraying activities.

A review of studies projecting the number of deaths from asbestos-related malignant mesothelioma in the United States indicated that the number of deaths during 1985–2009 would range from 620 to 3,270 annually (*9*). Based on an estimated 27.5 million workers with some exposure to asbestos during 1940–1972, a 1982 study estimated that the number of malignant mesothelioma deaths would rise to 3,060 annually by 2001–2005 (*4*). After 2005, mortality was projected to decrease but would continue for three decades. Based on asbestos consumption and malignant mesothelioma incidence data, it was estimated that the number of mesothelioma cases among males would peak during 2000–2004 (approximately 2,000 cases) and after that period, the number of mesothelioma cases was expected to decline and return to background levels by 2055 (*10*). The number of mesothelioma cases among females (approximately 560 in 2003) was projected to increase slightly over time. The results of the current study indicate an increase in the number of malignant mesothelioma deaths during 1999–2015. This discrepancy might be explained, in part, by the methodology of the projection studies, which were based on multiple assumptions including variations in the number of employed workers at risk, exposure levels and timing, and the linear dose–response relationship between asbestos exposure^¶¶¶^ and malignant mesothelioma. Moreover, additional persons who might have been exposed to asbestos and be at risk for malignant mesothelioma (e.g., family contacts of asbestos-exposed workers, persons exposed to naturally occurring asbestos, persons exposed to asbestos in surfacing materials or as fireproofing material in buildings) were not considered (*4*,*10*).

The findings in this report are subject to at least five limitations. First, information on exposure to asbestos or a specific work history was not available to assess the potential source of exposure. The industry and occupation listed on a death certificate might not be the industry and occupation in which the decedent's exposures occurred. Second, the state issuing a death certificate might not be the state or country in which the decedent's exposures occurred. Third, malignant mesothelioma did not have a discrete ICD code until the 10th revision of the ICD; thus, evaluation of mortality trends before 1999 was not possible. Fourth, some mesothelioma cases might not be included in this analysis because of misdiagnosis and the use of incorrect ICD-10 codes (*1*). Finally, information on decedents’ industry and occupation was available only for selected states of residence and years, and might not be nationally representative.

Despite regulatory actions and the decline in use of asbestos, the annual number of malignant mesothelioma deaths remains substantial. Effective asbestos exposure prevention strategies for employers recommended by OSHA and CDC’s National Institute for Occupational Safety and Health (https://www.cdc.gov/niosh/topics/asbestos/) are available. The continuing occurrence of malignant mesothelioma deaths underscores the need for maintaining asbestos exposure prevention efforts and for ongoing surveillance to monitor temporal trends.

**Summary**

**What is already known about this topic?**

Malignant mesothelioma is a neoplasm associated with inhalation exposure to asbestos fibers and other elongate mineral particles (EMPs). The median survival after malignant mesothelioma diagnosis is approximately 1 year. The latency period between the first exposure to asbestos fibers or other EMPs and mesothelioma development ranges from 20 to 71 years. Occupational exposure has occurred in industrial operations including mining and milling, manufacturing, shipbuilding and repair, and construction. Current occupational exposure occurs predominantly during maintenance and remediation of asbestos-containing buildings. The projected number of malignant mesothelioma deaths was expected to increase to 3,060 annually by 2001–2005, and after 2005, mortality was projected to decrease.

**What is added by this report?**

During 1999–2015, a total of 45,221 malignant mesothelioma deaths were reported, increasing from 2,479 (1999) to 2,597 (2015). Mesothelioma deaths increased for persons aged ≥85 years, for both sexes, persons of white, black and Asian or Pacific Islander race, and all ethnic groups. Continuing occurrence of malignant mesothelioma deaths in persons aged <55 years suggests ongoing inhalation exposure to asbestos fibers and possibly other causative EMPs.

**What are the implications for public health practice?**

Despite regulatory actions and decline in asbestos use, the annual number of malignant mesothelioma deaths remains substantial. Contrary to past projections, the number of malignant mesothelioma deaths has been increasing. The continuing occurrence of mesothelioma deaths, particularly among younger populations, underscores the need for maintaining efforts to prevent exposure and for ongoing surveillance to monitor temporal trends.

## Footnotes

* “Asbestos” is a term used for certain minerals that have crystallized in a particular macroscopic habit with certain commercially useful properties. “Asbestiform” is a term applied to minerals with a macroscopic habit similar to that of asbestos. https://www.cdc.gov/niosh/docs/2011-159/pdfs/2011-159.pdf.

† CDC WONDER. https://wonder.cdc.gov/.

§ Age-adjusted death rates were calculated by applying age-specific death rates to the 2000 U.S. Census standard population age distribution. https://wonder.cdc.gov/wonder/help/mcd.html#Age-Adjusted Rates.

¶ ICD-10 codes C45.0 (mesothelioma of pleura), C45.1 (mesothelioma of peritoneum), C45.2 (mesothelioma of pericardium), C45.7 (mesothelioma of other sites), and C45.9 (mesothelioma, unspecified). The death counts reported are the number of times each specific cause of death is mentioned in the record, or "any mention" of the specified cause of death. Up to 20 causes can be indicated on any single death certificate.

** https://webappa.cdc.gov/ords/norms.html.

†† PMR was defined as the observed number of deaths with malignant mesothelioma in a specified industry/occupation, divided by the expected number of deaths with malignant mesothelioma. The expected number of deaths was the total number of deaths in industry or occupation of interest multiplied by a proportion defined as the number of malignant mesothelioma deaths in all industries and/or occupations, divided by the total number of deaths in all industries/occupations. The malignant mesothelioma PMRs were internally adjusted by 5-year age groups, gender, and race.

§§ Underlying cause of death is defined as the disease or injury that initiated the chain of morbid events leading directly to death, or the circumstances of the accident or violence which produced the fatal injury.

¶¶ For 70 residents of these 23 states, deaths have occurred in states that did not provide the industry and occupation information to the National Institute for Occupational Safety and Health.

*** U.S. Department of Labor. OSHA. Asbestos. OSHA Standards (29 CFR 1910, 1915, and 1926). https://www.osha.gov/SLTC/asbestos/standards.html.

††† U.S. Environmental Protection Agency (EPA). Asbestos Laws and Regulations (https://www.epa.gov/asbestos/asbestos-laws-and-regulations) and EPA Asbestos Materials Bans: Clarification. May 18, 1999 (http://www.ewg.org/asbestos/documents/pdf/asb-bans2.pdf). On July 12, 1989, EPA issued a final rule banning most asbestos-containing products. In 1991, many aspects of this standard were set aside by the U.S. Fifth Circuit Court of Appeals. The following specific asbestos-containing products remain banned: flooring felt, rollboard, and corrugated, commercial, or specialty paper (58 Federal Register 58964). In addition, the regulation continues to ban the use of asbestos in products that have not historically contained asbestos, otherwise referred to as “new uses” of asbestos. Asbestos-containing product categories no longer subject to the 1989 ban include asbestos-cement corrugated sheet, asbestos-cement flat sheet, asbestos clothing, pipeline wrap, roofing felt, vinyl-asbestos floor tile, asbestos-cement shingle, millboard, asbestos-cement pipe, automatic transmission components, clutch facings, friction materials, disc brake pads, drum brake linings, brake blocks, gaskets, non-roofing coatings, and roof coatings.

§§§ In the United States, approximately 340 metric tons of asbestos were imported in 2016; nearly all asbestos was used by the chloralkali industry to manufacture semipermeable diaphragms. An unknown quantity of asbestos was imported within manufactured products, including brake linings and pads, building materials, gaskets, millboard, and yarn and thread, among others (Flanagan DM. U.S. Geological Survey, 2017, Mineral Commodity Summaries. Asbestos. https://minerals.usgs.gov/minerals/pubs/mcs/2017/mcs2017.pdf).

¶¶¶ Malignant mesothelioma can develop after short-term asbestos exposures of only a few weeks, and from very low levels of exposure. There is no evidence of a threshold level below which there is no risk for mesothelioma. The risk for mesothelioma increases with intensity and duration of asbestos exposure.
